# Reduction of oxidative cellular damage by overexpression of the thioredoxin *TRX2 *gene improves yield and quality of wine yeast dry active biomass

**DOI:** 10.1186/1475-2859-9-9

**Published:** 2010-02-12

**Authors:** Rocío Gómez-Pastor, Roberto Pérez-Torrado, Elisa Cabiscol, Joaquim Ros, Emilia Matallana

**Affiliations:** 1 Departamento de Bioquímica y Biología Molecular, Universitat de València, Valencia, Spain; 2 Departamento de Biotecnología, Instituto de Agroquímica y Tecnología de Alimentos, CSIC, Apartado de Correos, 73 Burjassot (Valencia), E-46100, Spain; 3 Departament de Ciències Mèdiques Bàsiques, IRBLleida, Universitat de Lleida, Spain

## Abstract

**Background:**

Wine *Saccharomyces cerevisiae *strains, adapted to anaerobic must fermentations, suffer oxidative stress when they are grown under aerobic conditions for biomass propagation in the industrial process of active dry yeast production. Oxidative metabolism of sugars favors high biomass yields but also causes increased oxidation damage of cell components. The overexpression of the *TRX2 *gene, coding for a thioredoxin, enhances oxidative stress resistance in a wine yeast strain model. The thioredoxin and also the glutathione/glutaredoxin system constitute the most important defense against oxidation. Trx2p is also involved in the regulation of Yap1p-driven transcriptional response against some reactive oxygen species.

**Results:**

Laboratory scale simulations of the industrial active dry biomass production process demonstrate that *TRX2 *overexpression increases the wine yeast final biomass yield and also its fermentative capacity both after the batch and fed-batch phases. Microvinifications carried out with the modified strain show a fast start phenotype derived from its enhanced fermentative capacity and also increased content of beneficial aroma compounds. The modified strain displays an increased transcriptional response of Yap1p regulated genes and other oxidative stress related genes. Activities of antioxidant enzymes like Sod1p, Sod2p and catalase are also enhanced. Consequently, diminished oxidation of lipids and proteins is observed in the modified strain, which can explain the improved performance of the thioredoxin overexpressing strain.

**Conclusions:**

We report several beneficial effects of overexpressing the thioredoxin gene *TRX2 *in a wine yeast strain. We show that this strain presents an enhanced redox defense. Increased yield of biomass production process in *TRX2 *overexpressing strain can be of special interest for several industrial applications.

## Background

The production of *Saccharomyces cerevisiae *biomass has become a powerful industry in the last years due to the increasing demand for modern winemaking practices, bread-manufacturing processes and also to its consumption as a dietary complement. Many selected natural yeast strains are now produced and commercialized as active dry yeast to be used as starters for must fermentation [1]. Several selection criteria for the choice of natural strains have been well established according to different aspects of the winemaking process, from the facilitation of specific stages to the improvement of wine organoleptic properties [2]. The search for wine yeast strains with innovative characteristics has traditionally relied on the isolation and screening for new yeast strains from grape and wine samples [3,4]. However, numerous research laboratories worldwide have succeeded in the generation, by genetic manipulation, of strains capable of improving processing efficiency, fermentative performance, and wine's sensory quality [1,5]. The commercial viability of genetically modified wine yeast strains has already been discussed [6].

During the last years, several studies have been carried out in order to analyze the complexity of the industrial biomass production process to use this knowledge as a tool for wine yeast strains improvement [7,8]. Previous stress gene marker analysis during bench-top trials of wine yeast biomass propagation demonstrated the induction of specific stress-related genes and enabled us to determine the environmental disturbances to which cells are dynamically exposed [7]. The data indicated that osmotic and, specially, oxidative stresses are the main two adverse conditions that *Saccharomyces cerevisiae *strains sense during the process. The relevance of oxidative stress for industrial yeasts performance including wine and brewing strains, and also different technological processes as brewing, wine making and biomass propagation has been pointed out in several studies [8-10]. The specific induction observed for the *TRX2 *gene in early stages of the biomass propagation process, especially under aeration conditions, suggested its involvement in the oxidative stress response. *TRX2 *gene codes for the yeast cytoplasmic thioredoxin 2, one of the most important redox controls together with glutathione/glutaredoxin system [11]. There are evidences pointing that both antioxidant systems are linked in the response against oxidative stress during specific biomass propagation, revealing novel overlapping roles between these two antioxidant systems [11,12]. Overexpression of the *TRX2 *gene in a wine yeast strain (T*TRX2*) produced an increase in the fermentative capacity in the biomass obtained at the end of the process [8]. The technological advantage of this improvement in the wine yeast properties encourage us to go far on the study of the oxidative stress response by different technical approaches. Despite the large accessible bibliography about oxidative stress mutants in laboratory yeast strains [13], very little is published about the effects of overexpression of oxidative stress genes [14], particularly in industrial yeasts.

The cellular response to reactive oxygen species involves a very complex network of biochemical mechanisms, from transcriptional control of gene expression to enzymatic repair of damaged cellular structures [12]. The transcriptional response affects a large number of genes participating in the different redox control and defense systems. In addition to the general stress response factors Msn2/4p, two specific transcriptional factors are mainly involved in reprogramming gene expression in response to oxidative injury, Yap1p and Skn7p [15]. Although they partially cooperate, the Skn7p factor controls only a subset of genes involved in the thioredoxin system, whereas the Yap1p factor is required for the induction of all the oxidative responsive genes [16]. Furthermore, other proteins, as Trx2p, have been implicated in the Yap1p-related oxidative response pathway [17].

Many genes induced under oxidative challenge code for antioxidant enzymatic activities which play important roles in cellular protection, both as ROS detoxifiers and regulators of the protein redox state [12]. Catalases, superoxide dismutases, and peroxidases are crucial to reduce the presence of ROS and several differentially regulated activities can be detected. In addition to these enzymatic detoxifiers, other proteins, such as thioredoxins and glutaredoxins, participate in the protection of protein activity against oxidative damage by repairing chemically modified proteins or by modulating the redox state of protein sulphydryl groups [11]. Several isoforms of these two types of thiol oxidoreductases are present in different subcellular compartments and act coordinately to maintain and recover full protein functioning. A complex interplay exists between these two main protein redox regulatory systems through the major redox buffer in eukaryotic cells, glutathione [18]. The redox potential of the GSH/GSSG couple determines the redox cellular state and is greatly influenced by ROS generation, both by addition of external oxidants or by metabolic leakage of electrons. The role of GSH in the connection of the two oxidoreductase systems has been pointed by the behavior of many different mutants and particularly by the increased GSH concentration and redox potential of the couple GSH/GSSG in a double *trx1trx2 *mutant [13,19].

Despite the presence of adaptative responses to oxidative stress, ROS accumulation can exceed the preventing and scavenging capacity of antioxidant defenses and cause damages on structural and functional cell components, such as nucleic acids, carbohydrates, lipids or proteins [20]. The cell membrane is a critical target for free radical attack, as lipid peroxidation can lead to cell leakage and death. High level of lipid peroxidation has been described as a common consequence of ROS accumulation, and also it has been related to the protective action of several antioxidant molecules [21]. Proteins are also major targets for ROS and different protein modification can lead to unfolding or alteration of protein structure [22]. Carbonylation is a well characterized, irreversible, and non-enzymatic modification of proteins which is most widely used as biomarker for oxidative damage of proteins [23].

In this work we found that the enhanced fermentative capacity produced by overexpression of thioredoxin 2 in a wine yeast strain correlated to an increased induction of several oxidative response genes, and also to increased activity of several ROS scavenging enzymes. Additionally, both total glutathione and the GSH/GSSG ratio were higher in the modified strain. In accordance to these effects on the protection mechanisms against oxidative stress, the T*TRX2 *strain displayed lower levels of molecular damage, and both lipid peroxidation and protein carbonylation were diminished. The improved response to oxidative stress caused by thioredoxin overexpression can explain the beneficial effects on fermentative performance, both in lab conditions and in microvinification experiments on natural musts, and does not affect negatively the analyzed oenological parameters of the produced wine.

## Results

### Improved performance of the T*TRX2 *strain during biomass propagation and winemaking

**Figure 1:**
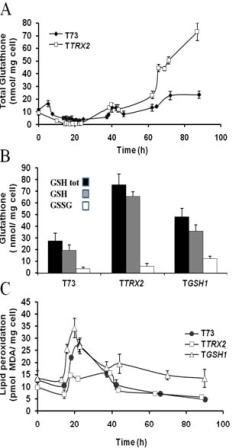
**Improved performance of T*TRX2 *strain in biomass production process**. (A) Biomass produced (continuous line) and oxygen saturation (discontinuous line) along bench-top trials of biomass propagation for T73 (black diamond), T*TRX2 *(white square) and *TGSH1 *(white triangle) strains by measuring OD_600 _from diluted samples. Average of three independent experiments and standard deviations are shown. (B) Fermentative capacity of yeast biomass collected at the end of the batch and fed-batch stages of growth in bench-top trials of ADY production. Biomass from wild-type T73 (*black bars*) and T*TRX2 *(*white bars*) were dehydrated until 8% moisture before performing the analysis. Data were normalized to the fermentative capacity of the batch sample from T73 strain. Average of three independent experiments and standard deviations are shown. Significantly different values compared to the control (p < 0.001) were marked by asterisk. (C) Sugar consumption profiles during microvinification experiments using natural Bobal must for T73 (closed symbols) and T*TRX2 *(open symbols) strains. The start of must fermentation was followed in detail during the first 6 hours for both strains T73 (closed symbol) and T*TRX2 *(open symbol). Averages were obtained from two independent experiments with three technical replicates for each one.

**Figure 2:**
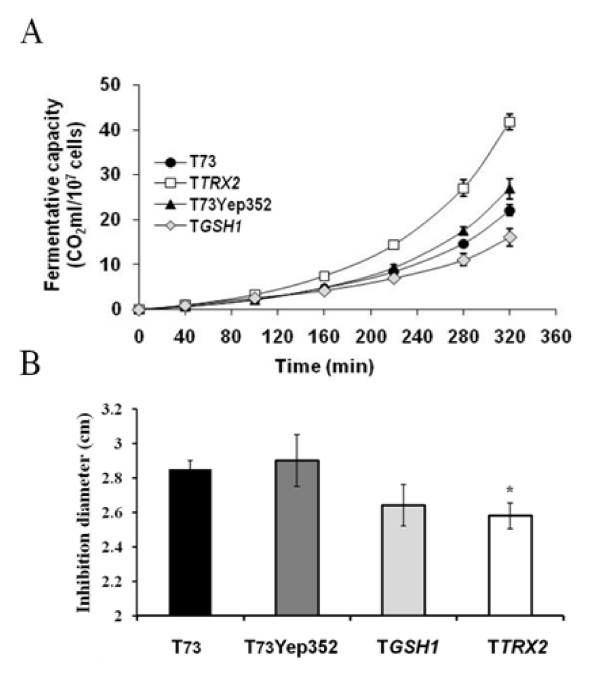
**Fermentative capacity and H_2_O_2 _growth inhibition assays for the improved T*TRX2 *strain compared to control T73 and T73Yep352 strains**. Fermentative capacity assays on YPGF medium for T73 (black circle), T*TRX2 *(white square), T73Yep352 (black triangle) and T*GSH1 *(white diamond) (A). Cells grown for 24 hours molasses medium were dehydrated and then assayed for CO_2 _production. (B) Determination of inhibition diameters produced with 10 μl of 30% H_2_O_2 _into paper discs on the growth of stationary cultures from T73, T73Yep352 and T*TRX2 *on YPD plates. Average of three independent experiments and standard deviations are shown. Significant differences were observed only for T*TRX2 *strain.

Bench-top trials of industrial wine yeast propagation were performed with the wine yeast model strain T73 and the genetically modified strain T*TRX2*, overexpressing *TRX2 *gene, and also in strain T*GSH1*, overexpressing *GSH1 *gene. Previous work [7,8] showed the suitability of using this simulation of the active dry yeast biomass production at laboratory scale, consisting of a fermentative batch step on molasses followed by a fed-batch step with low fed rate to ensure respiratory growth. At the end of the process, cells were dehydrated under hot air flow to obtain 8% final moisture ADY. The first indication of strain suitability for biomass production was the yield. When we compared ADY biomass yield for T73 (35.99 ± 3.34 g liter^-1^) and T*TRX2 *(54.78 ± 2.31 g liter^-1^) we observed a significant (p = 0.0106) increase of 52.2% (Figure 1A). In contrast, T*GSH1 *strain presented a reduced biomass yield (30.01 ± 2.01 g liter^-1^). However, the most important trait of ADY biomass is its performance in fermentation. To evaluate it, we first measured the fermentative capacity for the T*TRX2 *strain and the parental T73. The results (Figure 1B) showed a significant increase in fermentative capacity for the modified strain in biomass samples taken after the batch step and at the end of the process. Also, the modified strain displayed increased fermentative capacity during the fed-batch stage compared to the control strain. In order to discard artifactual effects related to the plasmid-based overexpression of the *TRX2 *gene, we also analyzed the fermentative capacity of T73 strain transformed with the empty vector, T73Yep352 strain, which was not significantly different (p = 0.12) to the control strain (Figure 2A). The behavior of this control strain was indistinguishable from the parental T73 strain in other growth and resistance tests, as growth on different liquid and solid media (see additional file 1) and in growth inhibition assays by hydrogen peroxide (Figure 2B). Also, the T*GSH1 *strain did not presented significant reduction of growth inhibition.

The winemaking performance of T73 and T*TRX2 *strains after ADY biomass production was assessed in controlled microvinification experiments on natural musts. As can be observed in Figure 1C, both strains presented similar sugar consumption profiles, especially at the end of microvinification, when sugar exhaustion occurred at the same time. However, the profiles are different at the beginning of the fermentation, when T*TRX2 *strain showed a significant reduction in the lag phase, starting the fermentation at least 4 hours before T73 strain. This phenotype was observed in three independent experiments, where no growth differences were observed between different precultures.

Several wine properties were also evaluated to asses the suitability of T*TRX2 *strain in winemaking (Table 1). Enological parameters (ethanol, glycerol, acetic acid and acetaldehyde), measured in the produced wine were no significantly different between the T*TRX2 *strain and the commercial T73 strain. However a noteworthy difference was observed between wines produced by the two strains in several aroma compounds. The levels of ethyl acetate, an undesirable flavor, and isoamyl alcohol are significantly decreased whereas the levels of desirable compounds as isoamyl acetate, succinate, caproate, caprylate and 2-phenylethanol acetate are significantly increased in wines from T*TRX2 *strain. Isobutanol, 2-phenylethanol, isobutyl acetate, 1-hexanol and hexil acetate presented similar levels in both strains.

**Table 1:** Wine parameters and final aroma concentration

	Strain		
	
	T73		T*TRX2*
*Enological parameters*		Mean ± SD (g/l)	
Glycerol	9.85 ± 0.151		9.6 ± 0.2
Acetic acid	0.55 ± 0.01		0.58 ± 0.02
Acetaldehyde	16.5 ± 0.43		20.37 ± 3.17
Ethanol	117.9 ± 10.2		94.32 ± 6.85
*Secondary aroma compounds*		Mean ± SD (mg/l)	
*Ethyl acetate	60.96 ± 7.03		22.83 ± 9.53
Isobutanol	59.76 ± 1.63		49.12 ± 5.71
**Isoamyl alcohol	180.66 ± 6.56		79.97 ± 1.56
2-phenylethanol	26.57 ± 6.16		44.75 ± 9.55
Isobutyl acetate	nd		0.045 ± 0.002
1-Hexanol	0.33 ± 0.06		0.19 ± 0.04
*Isoamyl acetate	1.14 ± 0.16		1.6 ± 0.02
*Caproate	0.16 ± 0.007		0.231 ± 0.006
Hexil acetate	0.016 ± 0.003		0.028 ± 0.001
*Succinate	0.069 ± 0.012		1.07 ± 0.027
*Caprylate	0.495 ± 0.028		0.81 ± 0.073
*2-phenylethanol acetate	0.27 ± 0.068		0.49 ± 0.025

Statistical significant with p-value *: p < 0.05 or **:p < 0.01; nd: below detection limits

### T*TRX2 *strain displays increased expression of oxidative stress related genes during the biomass production process

**Figure 3:**
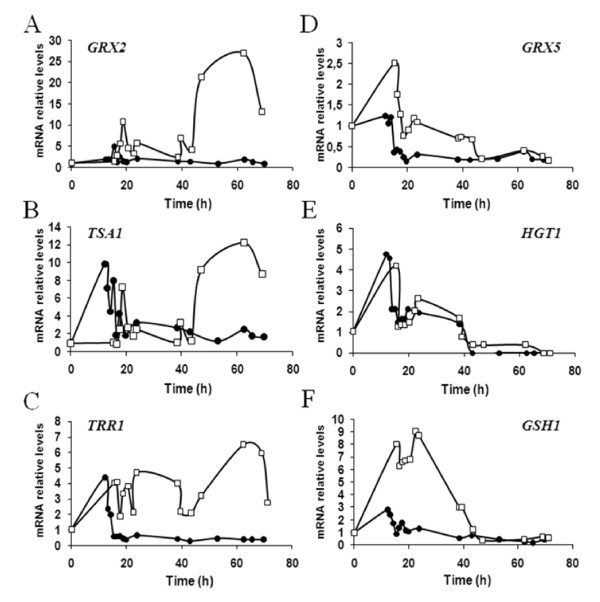
**Analysis of oxidative stress gene markers along biomass propagation bench-top trials**. mRNA relative levels for: (A) *GRX2 *(cytoplasmic glutaredoxin 2), (B) *TSA1 *(thioredoxin peroxidase), (C) *TRR1 *(thioredoxin reductase), (D) *GRX5 *(mitochondrial glutaredoxin), (E) *HGT1 *(high affinity glutathione transporter), and (F) *GSH1 *(γ-glutamylcysteine synthetase). Data from three independent experiments were normalized to 28S rRNA levels and to the probe-specific radioactivity. Results from one representative experiment are showed for T73 (*closed symbols*) and T*TRX2 *(*open symbols*).

In a previous study we described the transcriptional response against oxidative stress after glucose consumption (~15 h) during the batch phase of the ADY biomass propagation process [8]. As the *TRX2 *gene has been implicated in the regulation of the transcriptional response to oxidative damage [17] we decided to study the expression of several oxidative stress related genes in the T*TRX2 *strain compared to T73 strain. We selected two genes related to thioredoxin system, coding for thioredoxin peroxidase (*TSA1*) and thioredoxin reductase (*TRR1*), and four genes related to glutathione/glutaredoxin system, coding for glutaredoxins (*GRX2*, *GRX5*), γ-glutamylcysteine synthetase (*GSH1*) and the high affinity glutathione transporter (*HGT1*, also known as *OPT1*). It has been shown that the genes *TSA1*, *TRR1*, *GRX2 *and *GSH1 *are induced in response to external oxidative stress but the genes *GRX5 *and *HGT1 *seem not to be induced under this condition [20]. The expression analysis of strain T*TRX2 *compared to T73 strain showed increased mRNA amounts for all genes except *HGT1 *(Figure 3). *GRX5 *and *GSH1 *genes were expressed in T73 during the batch phase and similar profiles were observed for the T*TRX2 *strain, although with increased mRNA levels. In contrast, *TSA1*, *TRR1 *and *GRX2 *showed not only difference in the mRNA levels between both strains but also changes in the expression profile as they were not expressed during the fed-batch phase in T73 whereas they were induced in T*TRX2 *strain.

### Increased activity of ROS scavenging enzymes in T*TRX2 *strain

**Figure 4:**
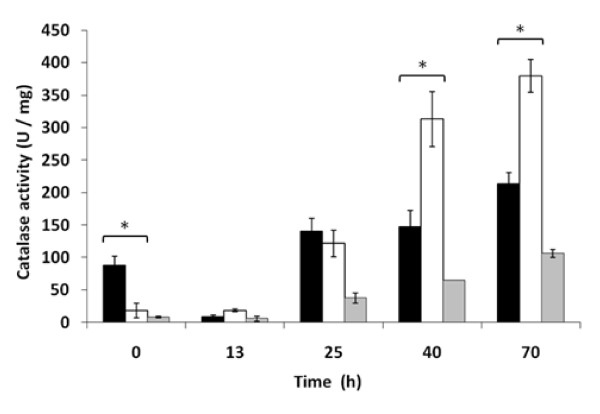
**Catalase activity along bench-top trials of biomass propagation for T73 (*black bars*), T*GSH1 *(grey bars) and T*TRX2 *(*white bars*) strains**. Average of three independent experiments and standard deviations are shown. Catalase activity was significantly different (p < 0.05) at time points marked by asterisks.

Thioredoxin and glutathione/glutaredoxin systems are the main cellular protection mechanisms under ROS injury but several antioxidant enzymatic activities, as catalases and superoxide dismutases (SOD), are also critical for a proper defense against cellular oxidation. As increased expression of thioredoxin and glutathione/glutaredoxin related genes was observed, we wondered if ROS scavenging antioxidant activities were also enhanced by *TRX2 *overexpression. Figure 4 shows catalase activity during biomass propagation for T73, T*TRX2 *and T*GSH1 *strains. In all the strains activity increased with biomass propagation time, achieving the maximal values at the last time point. However, T*TRX2 *strain showed significantly higher values of catalase activity during the fed-batch phase, when oxidative metabolism and low glucose concentration activate transcription of cytosolic catalase encoding gene *CTT1 *[15]. T*GSH1 *strain presented the lowest level of catalase activity.

**Figure 5:**
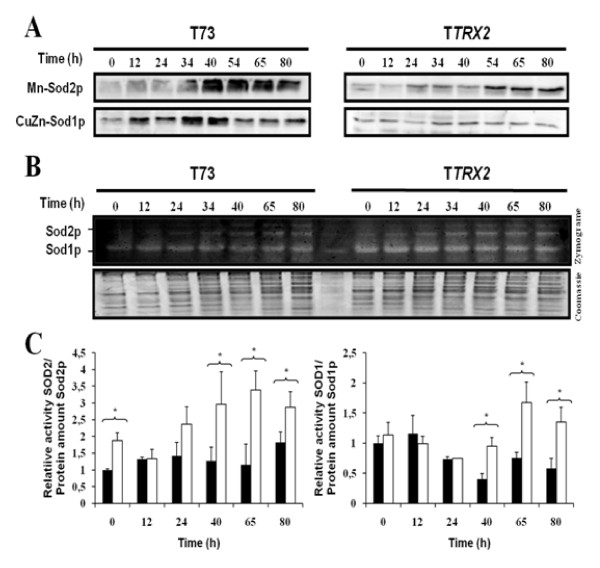
**Superoxide dismutase activity along bench-top trials of biomass propagation for T73 (*black bars*) and T*TRX2 *(*white bars*) strains**. Western blot analysis of cell extracts against Sod1p and Sod2p after electrophoresis under denaturing conditions (A). Panel B shows Zymogram obtained by native gel electrophoresis of cell extracts and SOD activity staining. (C) Mn-SOD and CuZn-SOD activity was calculated from zymogram density analysis and normalized to total protein amount from Coomassie stained gel. The specific activity was obtained as a result to normalized activity data to Sod1p and Sod2p protein amount from western blot analysis. In order to avoid technical errors as different exposure times, western blots and zymogram for both strains were carried out simultaneously in three independent experiments.

The activity of SOD1p and SOD2p isoenzymes was also followed during biomass propagation for T73 and T*TRX2 *strains. The amount of the corresponding protein was visualized by western blot and their activity was determined by zymogram analysis. The results showed in Figure 5 indicate that Sod1p isoform was present during both batch and fed-batch phases but mitochondrial Sod2p isoform was present mainly during fed-batch phase, likely due to high activation of respiratory metabolism. The accumulation profiles of Sod1p and Sod2p in T73 and T*TRX2 *strains (panel A) were similar but both isoforms were more abundant in wild type T73 strain. However, the activity detected in the zymogram analysis (panel B) revealed that, even with lower amounts of Sod1p and Sod2p, T*TRX2 *strain had significantly higher superoxide dismutase activity values, especially for Sod1p during the fed-batch phase (panel C). This can be a consequence of the decreased protein oxidation in the T*TRX2 *strain that permits increased Sod1p and Sod2p activities. In fact, it has been shown that increased carbonylation promoted by H_2_O_2 _oxidation can inactivate SOD among other activities [24]

### T*TRX2 *strain overaccumulates reduced glutathione during the fed-batch phase

**Figure 6:**
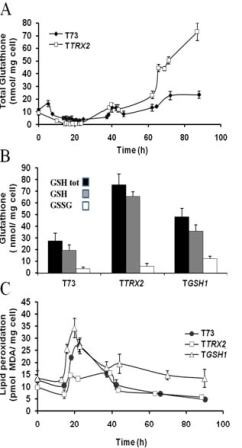
**Glutathione and lipid peroxidation analysis along bench-top trials of biomass propagation**. Total intracellular glutathione profile along biomass propagation bench-top trials for T73 (*closed symbols*) and T*TRX2 *(*open symbols*) strains (A). Glutathione levels at the end of the biomass propagation experiments for T73, T*TRX2 *and the T73 derivative overexpressing the *GSH1 *gene strain (T*GSH1*) (B). Total glutathione content (*black bars*), reduced GSH (*dark grey bars*) and oxidized GSSG (*white bars*) were determined. (C) Lipid peroxidation profile for T73 (black circle), T*TRX2 *(white square) and T*GSH1 *(white triangle) strains. The mean of three independent experiments and standard deviations are shown.

Differences between T*TRX2 *and T73 strains in the expression of the *GSH1 *gene, encoding the rate-limiting enzyme in glutathione synthesis [25], prompted us to determine the levels of glutathione during biomass propagation. As can be seen in Figure 6A, T*TRX2 *strain accumulated glutathione along the fed-batch phase, in contrast to T73 that kept this redox metabolite at basal levels. Furthermore, determinations of reduced (GSH) and oxidized (GSSG) glutathione forms in samples obtained at the end of fed-batch (Figure 6B) revealed an increased ratio GSH/GSSG in T*TRX2 *(13.12 ± 2.16) respect to T73 (7.60 ± 1.37). In order to check if the glutathione content increase observed for T*TRX2 *strain could be the main reason for the fermentative capacity improvement, we tried to implement these results by comparing with the modified strain (T*GSH1*). Glutathione levels significantly increased in T*GSH1 *strain respect to T73, however the GSH/GSSG ratio (4.37 ± 1.77) was lower compared to T73, indicating lower level of reduction equivalents available to protect against oxidative stress. In fact, T*GSH1 *strain showed decreased biomass yield compared to T73 (Figure 1A), decreased fermentative capacity (Figure 2A), similar resistance to H_2_O_2 _growth inhibition (Figure 2B), decreased catalase activity (Figure 4) and increased protein carbonylation (see additional file 2). These results suggest that GSH/GSSG ratio, rather than total glutathione content, is relevant for the improved performance of T*TRX2 *strain during the biomass production process.

### *TRX2 *gene overexpression causes diminished lipid peroxidation

Determination of lipid oxidation has been widely used to evaluate negative effects of ROS [21]. Lipid oxidation was measured during the biomass propagation experiments for the parental T73 and the genetically modified T*TRX2 *and T*GSH1 *strains (Figure 6C). The wild type industrial strain T73 showed a main peak of oxidation during the batch phase, when cells start to consume the ethanol produced during the initial fermentative phase. Strain T*GSH1 *showed a similar profile but lipid peroxidation was higher during the fed-batch phase, where catalase activity was lower than for T73. T*TRX2 *strain was more uniform and it did not show increased lipid peroxidation. This result supports the idea that T*TRX2 *strain has a better defense against the endogenous oxidative stress generated during the batch phase of the biomass propagation process.

The decrease in total glutathione levels in wild type T73 strain (Figure 6A) was coincident with the increase in lipid oxidation (Figure 6C). In fact, diminished glutathione level was already described in biomass propagation conditions for brewing yeasts [9]. Interestingly, T*TRX2 *strain displayed low glutathione levels from the beginning, probably due to a redox compensation as a consequence of the increased *TRX2 *gene levels. In contrast, it was more protected against lipid oxidation.

### Protein oxidation damage is reduced by *TRX2 *gene overexpression

**Figure 7:**
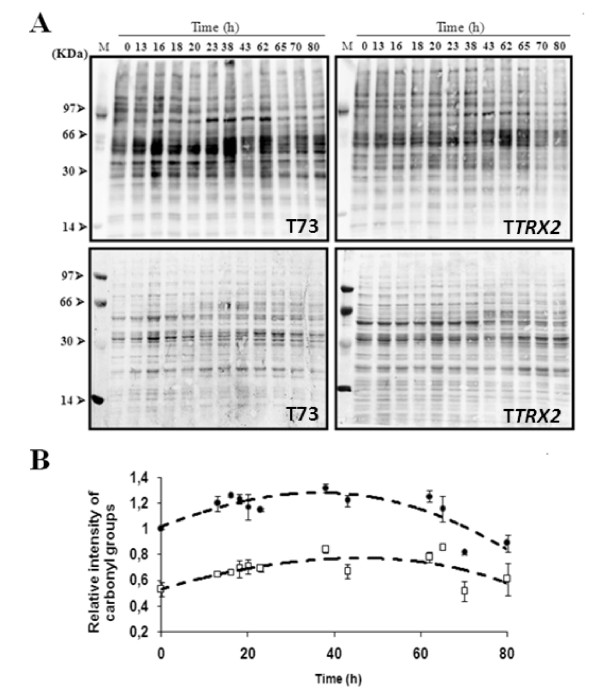
**Protein carbonylation along bench-top trials of biomass propagation**. Western analysis of oxidatively damaged proteins (top panels) and total protein stain (bottom panels) for T73 (left panels) and T*TRX2 *(right panels) strains (A). Panel B shows quantification of protein carbonyl content of T73 (black circles) and T*TRX2 *(open squares) data shown in panel A. Data was normalized to total protein in Coomassie stained gels. In order to avoid technical errors due to different exposure times, comparison between both strains was carried out in the same experiment. The mean of three independent experiments and standard deviations are shown. Protein carbonylation in samples from both strains between 0 and 65 h were significantly different with p < 0.01, and samples at 70 and 80 h were significantly different with p < 0.05.

In order to evaluate other biological effects of oxidation, samples along biomass propagation processes for both T73 and T*TRX2 *strains were taken and total protein carbonylation levels were analyzed by western blot using an anti-2,4-DNP antibody (Figure 7A, top panels). Quantification and normalization of these analyses (Figure 7B) revealed significant differences along the process for both strains. The relative levels of protein carbonylation in T73 strain varied from 1.3 ± 0.03 to 0.85 ± 0.02, with a maximal peaks at 38 h and a minimum at 70 hours. T*TRX2 *strains presented lower protein carbonylation levels over the whole experiment time course, ranging from 0.85 ± 0.01 to 0.51 ± 0.07, and maintaining the same time points for the maximal and minimal peaks. At the end of the process protein carbonylation decreased in both strains.

## Discussion

Studies by our group and others have found that oxidative stress response is critical during industrial yeast biomass propagation [7-9]. Therefore we designed several strategies of genetic modification in order to enhance the response of the commercial wine strain T73 against oxidative damage. Here we present the most promising one: the T*TRX2 *strain, carrying an overexpression of the thioredoxin-encoding gene *TRX2*, which shows a dual improvement. This strain is able to increase the produced biomass yield during cell propagation experiments and to increase the fermentative capacity after ADY biomass production that is observed as a "fast start" winemaking phenotype. Our results show that T*TRX2 *strain has an enhanced molecular oxidative stress response, and then it suffers a lower degree of oxidative damage on macromolecular components, such as lipids and proteins.

Optimized industrial yeast propagation processes are designed to produce the highest biomass yield by forcing the oxidative catabolism of molasses sugars. In the case of wine yeasts, the produced biomass is dehydrated to preserve the starter as ADY, due to its seasonal use for musts inoculation after grape harvest. The whole industrial process has critical impact on the quality of the produced biomass affecting viability, vitality and fermentation rate, and these parameters are determinant for yeast performance in winemaking. Previous results pointed out that the oxidative stress response of yeast cells is important during the biomass production process [8], probably because the respiratory metabolism and even fermentation [10] generates endogenous ROS. Furthermore, ROS generation during dehydration can also damage cell structures [26]. Our hypothesis was that a modified yeast strain showing enhanced oxidative stress defenses would improve their performance during the ADY biomass production process and also afterwards, in winemaking. Several modifications in genes related to the glutathione and thioredoxin redox systems were carried out in the commercial wine yeast strain T73 and the overexpression of *TRX2 *gene was selected for further studies. Simulations of the industrial biomass propagation process were developed at laboratory scale and they showed that this modified strain displays higher fermentative capacity during batch and fed-batch stages, and also that it produces higher biomass yield at the end of the process. These properties of the strain T*TRX2 *can have high economical impact for yeast biomass producers. Furthermore, when ADY biomass was used as starter for microvinification experiments in natural must, the lag phase of fermentations conducted by T*TRX2 *strain was significantly reduced compared to T73. This "fast starting" phenotype is a valuable character because it can help rapid microbial stabilization at the beginning of grape must fermentation and ensure the domination of the selected strain over natural strains. Furthermore, it presents increased content of aroma compounds, probably due to a better performance of less oxidized enzymes implicated in their synthesis.

The beneficial effect of thioredoxin increased dosage on oxidative stress defense has been extensively described in different organisms and circumstances. Overexpression of human *TRX1 *in transgenic mice (TRX1-Tg) has been reported to provide a protective activity against oxidative stress-related disorders such as post-ischaemic reperfusion injury in the brain [27] kidney [28], diabetic nephropathy [29] and adriamycin-induced cardiotoxicity [30]. Beneficial effects related to oxidative response has been also described in plants [31]. In yeasts, *TRX2 *was found to be essential for a proper oxidative response against hydroperoxides [32]. The oral administration of thioredoxin has been proposed in clinical and functional foods applications due to its antioxidant, anti-inflammatory and antiallergenic properties [33,34]. From this point of view, the strain T*TRX2 *will be valuable for the industrial production of thioredoxin and also for production of functional foods.

An effort to understand the physiology of the T*TRX2 *strain at molecular level and explain its efficiency improvement has been made. Experimental data obtained in laboratory strains adding external oxidants support that *TRX2 *is involved in Yap1p reduction and inactivation [35]. But this mechanism is not completely understood because *trx*^- ^mutant strains present normal inactivation after diamide treatment. Accordingly to our results in industrial strains, overexpression of *TRX2 *causes increased responses in oxidative defenses in the industrial modified strain T*TRX2*. In fact, Yap1-regulated genes, and also other oxidative stress related genes, showed increased mRNA levels in T*TRX2 *strain. These results can be explained in several ways. First, our experiments were developed with industrial strains that show important differences in gene expression compared with laboratory strains [36]. Second, Yap1-mediated regulation was studied in laboratory conditions as a response to external oxidants. In contrast, the transcriptional responses observed in this study were likely due to internal production of ROS derived from respiratory metabolism. A possible explanation for the behavior of the T*TRX2 *strain is that increased levels of Trx2p alter the NADPH/NADP^+ ^ratio, as these molecules participate in the maintenance of the thioredoxin redox state. Furthermore, increased GSH/GSSG ratio can alter NADPH/NADP^+ ^balance since glutathione reductase enzyme need NADPH to reduce GSSG to GSH. Changes in this redox buffer would be sensed as an oxidative alteration and it would promote stronger oxidative stress responses.

Several data support that T*TRX2 *strain has enhanced defense against oxidative stress. It shows increased mRNA levels of oxidative stress related genes, and also increased glutathione content, increased GSH/GSSG ratio and increased activities of antioxidant enzymes, such as superoxide dismutases and catalase. All these elements would produce an enhanced protection against ROS-mediated oxidation. In fact, here we present consistent data supporting that T*TRX2 *strain displays lower oxidation damages than the control strain. Lipid oxidation is drastically increased in the control strain during ethanol respiration in the batch phase (20-30 h), probably due to oxidative metabolism and endogenous ROS generation. However, the T*TRX2 *strain presents much lower level of lipid oxidation. Interestingly, glutathione levels change during biomass production process. A possible explanation for the glutathione decrease in the wild type strain T73 can be its use in glutathionylation reactions. This protein modification it's important in different organisms for the correct function of many proteins, including glycolytic enzymes, in response to oxidative stress conditions [37]. The low glutathione levels in the T*TRX2 *strain during first hours of fermentation could be due to a higher degree of glutathionylation events. This effect would be in agreement with the increased expression of oxidative stress genes, supporting that T*TRX2 *overexpression generates a cellular signal that activates responses against oxidation.

The level of cellular oxidation was also followed by determination of carbonyl groups in proteins. Strain T*TRX2 *presents much lower level of protein carbonylation during the biomass propagation process than the control T73 strain, corroborating that thioredoxin overaccumulation protects against oxidation events. Protein oxidation leads to altered protein structure and function [22] so negative effects on cell physiology are expected when cells suffer ROS attacks. The diminished protein oxidation observed for the T*TRX2 *strain is then consistent with its better performance in biomass propagation trials and could be related to a better preserved activity of specific metabolic or detoxifying enzymes. Also, our previous work showed that specific proteins involved in sugar fermentation were heavily damaged in oxidative conditions like chronological or replicative aging [38].

## Conclusions

In this study we have shown the beneficial effects of enhancing stress redox response by overexpressing a cytosolic thioredoxin in a wine yeast strain. The strain T*TRX2 *that overexpresses the *TRX2 *gene produces higher biomass in an ADY biomass industrial production process modelization at laboratory scale. Furthermore, T*TRX2 *yeast biomass presents a better quality as indicates its increased fermentative capacity and decreased lag phase. It's also able to produce wines with enhanced aroma compounds in microvinification experiences.

Enhanced performance of T*TRX2 *strain is a consequence of its increased defense against redox stresses. In fact, it presents increased levels of antioxidant glutathione and reduced levels of proteins and lipids oxidation and increased levels of ROS scavenging enzymes Sod1p and Sod2p. The increased defense against redox stress of T*TRX2 *can be just a consequence of the increased content of cytosolic thioredoxin due to its antioxidant properties. Our data points to other side effects derived from the implication of *TRX2 *in gene transcription regulation since T*TRX2 *strain presented increased transcription of redox genes. However, the fact that this regulation is not completely understood and the complexity of the relations between thioredoxin and glutathione systems make difficult to establish the specific role of *TRX2 *in T*TRX2 *strain.

We think T*TRX2 *strain can be very valuable to be used as a cell factory for added-value products or for several industrial applications as biomass production and production of flavored wines.

## Materials and methods

### Yeast strains, plasmids and cultivation conditions

*S. cerevisiae *industrial strain T73 (CECT1894) is a natural diploid strain isolated from Alicante (Spain) musts [39] and has been commercialized by Lallemand Inc. (Montreal, Canada). This strain has been previously used in several studies [7,8,35,40,41] and has proven to be a good wine yeast model.

The YEp-*TRX2 *plasmid was obtained by subcloning a 0.7 kb *Eco*RI fragment containing the yeast *TRX2 *gene into the episomal yeast plasmid YEp352 vector. T*TRX2 *strain [8] is a genetically modified T73 strain obtained by transformation of a *ura*^- ^T73 derivate [42] with YEp-*TRX2 *following the lithium acetate procedure as modified by [43]. The empty YEp352 vector was also introduced in the T73 *ura*^- ^strain as a control of its influence in growth conditions and oxidative stress experiments. The T*GSH1 *strain was obtained transforming T73 *ura*^- ^with a YEp352-*GSH1 *plasmid, kindly provided by Dr A.K. Bachhawat.

Precultures for industrial biomass propagation experiments were prepared in YPD liquid medium (1% Yeast extract, 2% Peptone, 2% Glucose) and were incubated at 30°C with shaking (250 rpm) during 12 h. YPD plates (1% Yeast extract, 2% Peptone, 2% Glucose, 2% Agar) were used for growth analysis in laboratory conditions. The liquid medium YPGF (1% Yeast extract, 2% Peptone, 10% Glucose, 10% Fructose) was used to test the fermentative capacity of cells produced in industrial conditions.

Molasses medium (diluted to 60 g of sucrose liter^-1 ^for batch phase or 100 g of sucrose liter^-1 ^for fed-batch phase) was supplemented with 7.5 ml of (NH_4_)_2_SO_2 _liter^-1^, 3.5 g of KH_2_PO_4 _liter^-1^, 0.75 g of MgSO_4_7H_2_O liter^-1^, 10 ml of vitamin solution liter^-1^, and 1 ml of antifoam 204 (Sigma, St. Louis, Mo.) liter^-1^. Molasses and mineral solutions were autoclaved separately. The vitamin solution containing 50 mg of D-biotin liter^-1^, 1 g of calcium pantothenate liter^-1^, and 1 g of thiamine hydrochloride liter^-1 ^was filter sterilized (0.2-μm pore size) prior to use in the molasses medium.

### Industrial propagation conditions

Biomass propagation experiments were designed with two growth stages, batch and fed-batch, in a bioreactor BIOFLO III (NBS, New Jersey, USA), and technical parameters (agitation, pH and fed rate) were established as previously described [7,8]. The bioreactor containing 2 liter of sterilized molasses medium at pH 4.5 was inoculated to an initial optical density of 0.05 (OD_600 _= 0.05) from overnight YPD precultures. During batch phase cells consumed all the sucrose present into the medium using a fermentative metabolism. When sucrose was finished (12-15 h), cells changed its metabolism to a respiratory metabolism allowing the consumption of the ethanol produced, until approximately 40 h of the process. During this phase pH was allowed to freely vary between 4 and 5. When the ethanol was finished, the fed-batch phase started feeding the reactor continuously with molasses medium by a type 501 peristaltic pump (Watson-Marlow, Falmouth, United Kingdom) at the desired flow rate, avoiding fermentative metabolism. During the fed-batch phase, the reactor pH was maintained at 4.5 by the automatic addition of 42.5% H_3_PO_3 _or 1 M NaOH. Dissolved oxygen, measured with an electrode (Mettler-Toledo), was maintained above 20% by a PID control system that allowed the automatic modification of the agitation speed between the range limits of 300 to 500 rpm. Four independent experiments were carried out for each T73 and T*TRX2 *strains.

### Analysis and quantification of mRNA

Total yeast RNA was obtained from yeast cells (50 mg) with the hot phenol method [44]. Samples were analyzed by electrophoresis in formaldehyde-containing agarose gels and by Northern blotting. Specific primers for PCR synthesis of DNA probes are shown in Table 2. Probes were labeled by random priming (High Prime, Roche) using [α^32^P]dCTP (Amersham). High stringency conditions were used both for hybridization and washes. mRNA quantification was carried out by direct measurement of radioactivity on the filters with an Instant Imager FLA-5000 and the Image Gauge software (FujiFilm, USA). Sample data were normalized to gel stained 28S rRNA levels and to the gene probe specific radioactivity. An internal control gene was used for consecutive membrane hybridizations. Gene expression experiments were performed in triplicate.

**Table 2:** Genes and primers used for the amplification of DNA probes

Probe	Primer	Sequence (5'-3')	Probe length (bp)
*GRX2*	*GRX2*-1	GTATCCCAGGAAACAGTTGC	945
	*GRX2*-2	GTTTCCAAATCGCTGTTACC	
*GRX5*	*GRX5*-1	CATAAGGTCATTTTCCCCC	407
	*GRX5*-2	CTTCTTCAGGTACCAATGCC	
*TSA1*	*TSA1*-1	CAAGTTCAAAAGCAAGCTCC	461
	*TSA1*-2	TCAACCAATCTCAAGGCTTC	
*TRR1*	*TRR1*-1	ATGAAGGTATGATGGCGAAC	779
	*TRR1*-2	ATCCTGAACATCACCAGCAG	
*GSH1*	*GSH1*-1	CCCGATGAAGTCATTAACA	945
	*GSH1*-2	GGAAAAGGTCAAAATGCT	
*HGT1*	*HGT1*-1	GGACAATCATCGGAAGCTG	817
	*HGT1*-2	GAGCTTGCAGGGTTGACGAC	

### Biomass dehydration and measurement of fermentative capacity

Yeast biomass was dehydrated under air flux in an oven at 30°C until approximately 8% relative humidity and kept at room temp for few days. For determination of fermentative capacity, dehydrated cells were inoculated (10^7 ^cells/ml) in YPGF and incubated at 30°C and 65 rpm. CO_2 _production (in ml) was measured every 20 min for 6 h in a Chittick instrument (American association of cereal Chemist, 12-10). After sample normalization to cell number, fermentative capacity is expressed as ml of CO_2 _produced per 10^7^cells.

### Determination of glutathione

Glutathione was determined as described previously [45]. Collected cells (100 mg) were washed twice with phosphate-buffer saline (PBS, pH 7.4) and suspended in 1 ml ice-cold 8 mM HCl, 1.3% (w/v) 5-sulphosalicylic acid. Cells were broken by vortexing at 4°C with 0.5 g of glass beads in four series of 1 min alternated with 1 min incubation on ice. Cell debris and proteins were pelleted in a microcentrifuge for 15 min (13000 rpm at 4°C), and supernatants were used for glutathione determination. For total GSH determination, supernatant was used directly in 200 μl of total volume reaction while GSSG determination was carried out in the same volume reaction with 2 μl of 1 M 2-vinyl-piridine for 1 h at room temperature. Reduced GSH values were obtained as the difference between total glutathione and oxidized glutathione. Glutathione levels are expressed as nmol per mg of cells.

### Quantification of lipid peroxidation

Quantification of lipid peroxidation was carried out by reaction of thiobarbituric acid with the malondialdehide (MDA) product of oxidized fatty acid breakage [46] Cells (50 mg) were collected, washed twice with distilled water and then extracted by vortexing with 0.3 g glass beads in 0.5 ml of 50 mM sodium phosphate buffer, pH 6.0, 10% trichloroacetic acid (TCA), in three series of 1 min alternated with 1 min incubation on ice. After centrifugation at 13000 rpm for 10 min, 300 μl of supernatants were mixed to 100 μl of 0.1 M EDTA and 600 μl 1% thiobarbituric acid in 0.05 M NaOH, and then incubated at 100°C 15 min. After cooling on ice and centrifugation to eliminate precipitates, malondialdehide was measured by the absorbance at 535 nm. The molar absorptivity of MDA (1.56 × 10^5 ^M^-1 ^cm^-1^) was used to express lipid peroxidation levels as pmoles of MDA per mg of cells.

### Quantification of protein carbonylation

Cells were collected, washed twice in distilled water and then extracted by vortexing with 0.4 g glass beads in 0.5 ml of extraction buffer (25 mM imidazol, 2 mM EDTA pH 7.0) and a mix of protease inhibitors (200 μM phenylmethylsulfonyl fluoride (PMSF), 20 μM TPcK, 200 μM pepstatin A). Protein concentration was determined by the Bradford assay [47] and all samples were diluted in SDS 6% using the Bio-Rad Protein Assay following manufacturer instructions. Protein carbonylation in crude extracts was measured by dinitrophenilhydrazine (DNPH) derivatization and western immunodetection of protein-bound 2,4-dinitrophenylhydrazones [48]. The anti-2,4-dinitrophenol antibody (DAKO Ref. V0401) was used at a 1/4000 dilution and the secondary antibody (goat anti-rabbit HRP conjugated, Amersham) was used at a 1/25000 dilution. Signals in blots were visualized using Lumigen TMA-6 (Amersham) and images were analyzed using Bio-Rad Lumicapt software.

### Determination of antioxidant enzyme activities

Cell extracts for enzymatic determinations were prepared using glass beads and assayed as described for catalase [49] and superoxide dismutase [50] activities. Mn-SOD and CuZn-SOD activities were analyzed after separating cell lysates in native acrylamide gel electrophoresis. Activity stained bands were measured with a densitometer and the relative density calculated by the Quantity One software (Bio-Rad). Data were normalized to total loaded protein from Coomassie blue stained gels and to the amount of Sod1p and Sod2p proteins from western blot assays. Western Blots were performed in triplicate. To analyze the amounts of Sod1p and Sod2p, cell extracts were separated in SDS-polyacrylamide gels and transferred to polyvinylidene difluoride membranes. Antibodies against superoxide dismutases were purchased from Stress-Gene (SOD-111, Mn-SOD) and Chemicon (AB1237, CuZn-SOD). A secondary HRP-conjugated anti-rabbit antibody was used for detection. Image acquisition was performed in a ChemiDoc CCD camera (Bio-Rad). Three independent experiments were carried out.

### Microvinification experiments

For microvinification experiments, natural must from Bobal red grapes (Requena, Spain) was sterilized with 0.2% (vol/vol) dimetyl dicarbonate for 48 h at 4°C to allow decomposition. 0.2 liter glass bottles were completely filled and capped to reproduce the anaerobic conditions of wine fermentation. Musts were inoculated (5 × 10^6 ^cells/ml) of rehydrated air-dried biomass (24 h, 30°C) from 24 h grown yeast cultures on molasses. Bottles were incubated at 28°C with gentle shaking (125 rpm) without aeration. Culture growth was followed by measuring optical density at 600 nm (OD_600_).

### Determination of reducing sugar concentration, ethanol, and enological parameters

Reaction with DNS (10 g liter^-1 ^3,5-dinitrosalicylate, 16 g liter^-1 ^sodium hydroxide, 300 g liter^-1 ^potassium sodium tartrate 4-hydrate), and measurement of the developed color (as OD at 540 nm) was used for quantification of reducing sugars, with a glucose standard curve between 0 and 2 g liter^-1^. Alcoholic grade (g liter^-1^) was determined by the alcohol dehydrogenase (ADH) enzymatic assay, with ethanol and NAD^+ ^as substrates, by measuring the NADH produced (OD at 340 nm) and using a standard curve 0 and 0.15 mM ethanol. Other enological parameters (acetic acid, glycerol and acetaldehyde) were quantified by automated enzymatic determination (ECHO; Logotech).

### Gas-liquid chromatography

The gas chromatographic analysis of volatile compounds was carried out using a Hewlett-Packard capillary gas chromatograph, model 5890 series II, controlled with a 3365 Chemstation (flame ionization detector; Supelcowax 10 [Supelco, Inc., Bellafonte, Pa.] fused-silica capillary column [30 m by 0.25 mm, 0.25 μm film]). Final microvinification samples (1.5 ml) were analyzed using 0.005% (w/v) 2-heptanone as internal standard. Each volatile compound was quantified within each sample as a dimensionless number corresponding to the area of the chromatographic peak divided by the area of the internal standard.

### Statistical analysis

Sample averages were compared to measure significance of the difference using Fisher Exact t-Test online tool SISA (Simple Interactive Statistical Analysis). P-values below 0.05, 0.01 or 0.001 (labeled as *, ** or *** respectively) were used.

## Abbreviations

ADH: Alcohol dehydrogenase; DNPH: 2,4-dinitrophenylhydrazine; GSH: Reduced glutathione form; GSSG: Oxidized glutathione form; HRP: Horseradish peroxidase; OD: Optical density; PBS: Phosphate buffered saline; PMSF: Phenylmethylsulfonyl fluoride; ROS: Radical oxygen species; SOD: Superoxide dismutase; TCA: Trichloroacetic acid; TPcK: N-tosyl-L-phenylalanylchloromethyl ketone dinitrophenilhydrazine.

## Competing interests

The authors declare that they have no competing interests.

## Authors' contributions

RGP carried out most of the experiments and contributed to the writing of the manuscript. RPT initiated the project, assisted with conception, data interpretation, statistical analysis and draft the manuscript. EC and JR assisted with the experimental design of oxidative stress experiments. EM conceived of the study, participated in its design, and contributed to the writing of the manuscript. All authors read and approved the final manuscript.

## Supplementary Material

Additional file 1 **Growth parameters for the improved T*TRX2 *strain compared to control T73 and T73Yep352 strains**. Growth analysis for T73 (black circle), T*TRX2 *(black triangle) and T73Yep352 (white square) strains on YPD plates (A) or liquid (B) medium. No significant differences were observed. Plates were spotted with 5 μl of exponential cultures equaled to OD = 0.1 and 1/5 serial dilutions. Click here for file

Additional file 2 **Protein carbonylation along bench-top trials of biomass propagation for T73 and T*GSH1 *strain**. Western analysis of oxidatively damaged proteins (top panels) and total protein stain (bottom panels) for T73 (left panels) and T*GSH1 *(right panels) strains (A). Panel B shows quantification of protein carbonyl content of T73 (black circles) and T*GSH1 *(open squares) data shown in panel A. Data was normalized to total protein in Coomassie stained gels. In order to avoid technical errors due to different exposure times, comparison between both strains was carried out in the same experiment. The mean of three independent experiments and standard deviations are shown. Protein carbonylation in samples from both strains between 0 and 65 h were significantly different with p < 0.01, and samples at 70 and 80 h were significantly different with p < 0.05. Click here for file
