# Correction: reduction of oxidative cellular damage by overexpression of the thioredoxin TRX2 gene improves yield and quality of wine yeast dry active biomass

**DOI:** 10.1186/1475-2859-11-31

**Published:** 2012-03-05

**Authors:** Rocío Gómez-Pastor, Roberto Pérez-Torrado, Elisa Cabiscol, Joaquim Ros, Emilia Matallana

**Affiliations:** 1 Departamento de Biotecnología, Instituto de Agroquímica y Tecnología de Alimentos, CSIC, Apartado de Correos, 73 Burjassot (Valencia), E-46100, Spain; 2 Departament de Ciències Mèdiques Bàsiques, IRBLleida, Universitat de Lleida, Spain; 3 Departamento de Bioquímica y Biología Molecular, Universitat de València, Valencia, Spain

## Correction

Following publication of this work [1] we have noticed a production error in the article. Figure 1 in the original version showed incorrect results, with graphs having been duplicated in error from another figure. The correct results for Figure 1 are shown below.

**Figure 1:**
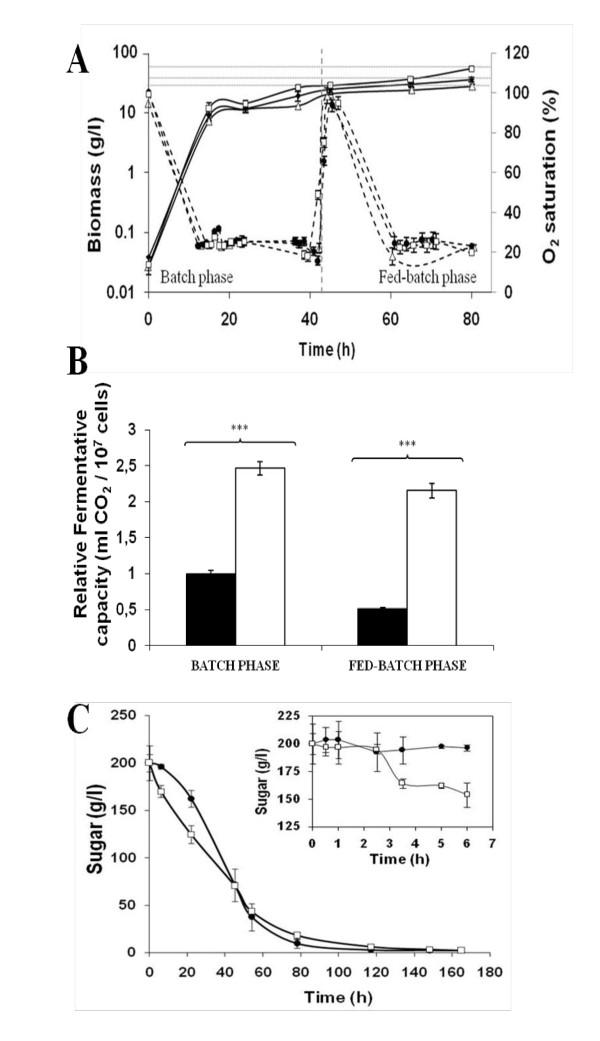
**Improved performance of T*TRX2 *strain in biomass production process**. (A) Biomass produced (continuous line) and oxygen saturation (discontinuous line) along bench-top trials of biomass propagation for T73 (black diamond), T*TRX2 *(white square) and *TGSH1 *(white triangle) strains by measuring OD_600 _from diluted samples. Average of three independent experiments and standard deviations are shown. (B) Fermentative capacity of yeast biomass collected at the end of the batch and fed-batch stages of growth in bench-top trials of ADY production. Biomass from wild-type T73 (*black bars*) and T*TRX2 *(*white bars*) were dehydrated until 8% moisture before performing the analysis. Data were normalized to the fermentative capacity of the batch sample from T73 strain. Average of three independent experiments and standard deviations are shown. Significantly different values compared to the control (p < 0.001) were marked by asterisk. (C) Sugar consumption profiles during microvinification experiments using natural Bobal must for T73 (closed symbols) and T*TRX2 *(open symbols) strains. The start of must fermentation was followed in detail during the first 6 hours for both strains T73 (closed symbol) and T*TRX2 *(open symbol). Averages were obtained from two independent experiments with three technical replicates for each one.
