# Supplementary figures and images for: Reduction of oxidative cellular damage by overexpression of the thioredoxin TRX2 gene improves yield and quality of wine yeast dry active biomass
Source: Microb Cell Fact. 2010 Feb 12;9:9. doi: 10.1186/1475-2859-9-9 (PMC2835662; doi:10.1186/1475-2859-9-9)

## Slide 1
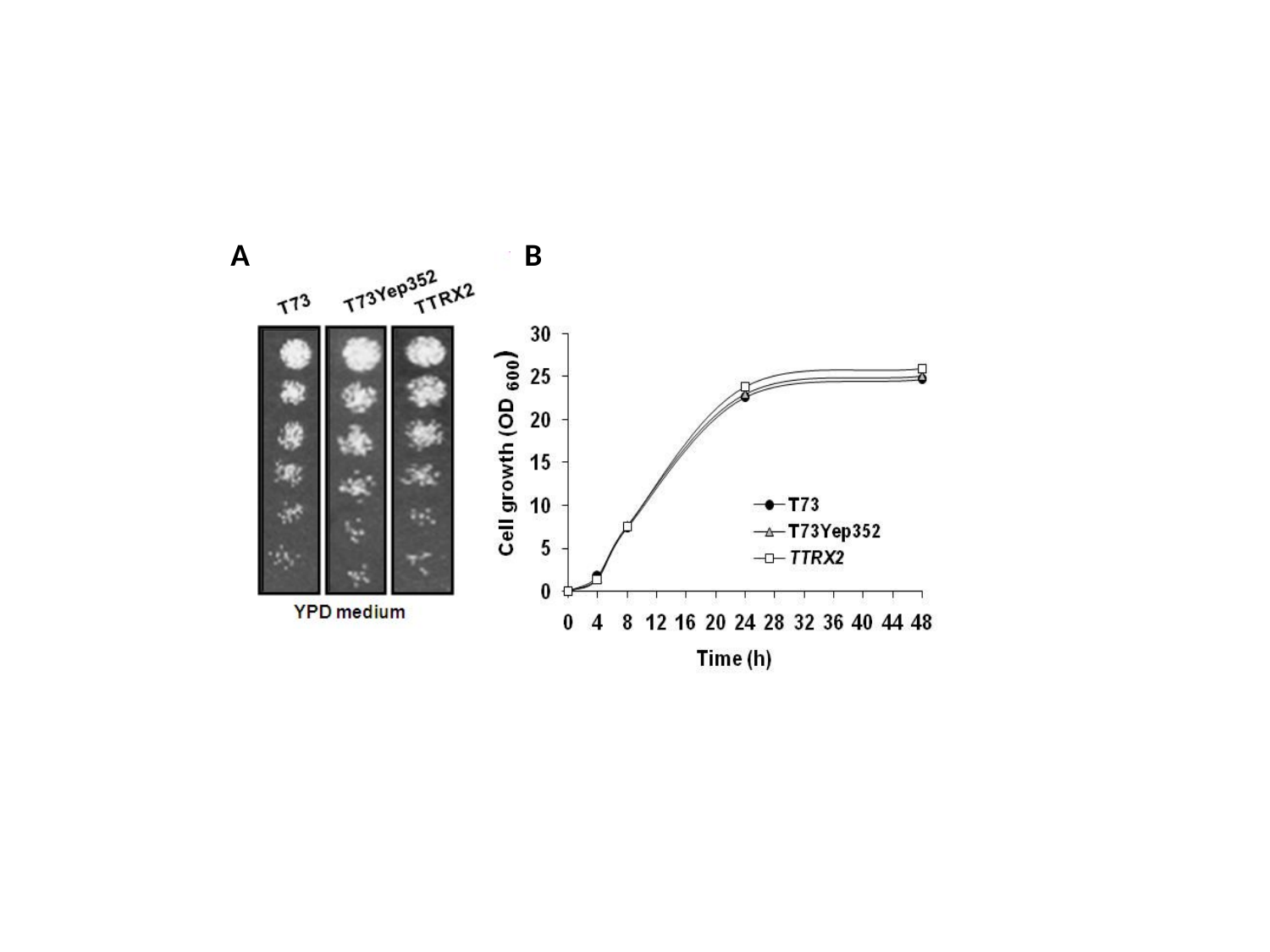

A
B

Supplement: Additional file 1 — Growth parameters for the improved TTRX2 strain compared to control T73 and T73Yep352 strains. Growth analysis for T73 (black circle), TTRX2 (black triangle) and T73Yep352 (white square) strains on YPD plates (A) or liquid (B) medium. No significant differences were observed. Plates were spotted with 5 μl of exponential cultures equaled to OD = 0.1 and 1/5 serial dilutions. [file 1475-2859-9-9-S1.PPT]

## Slide 1
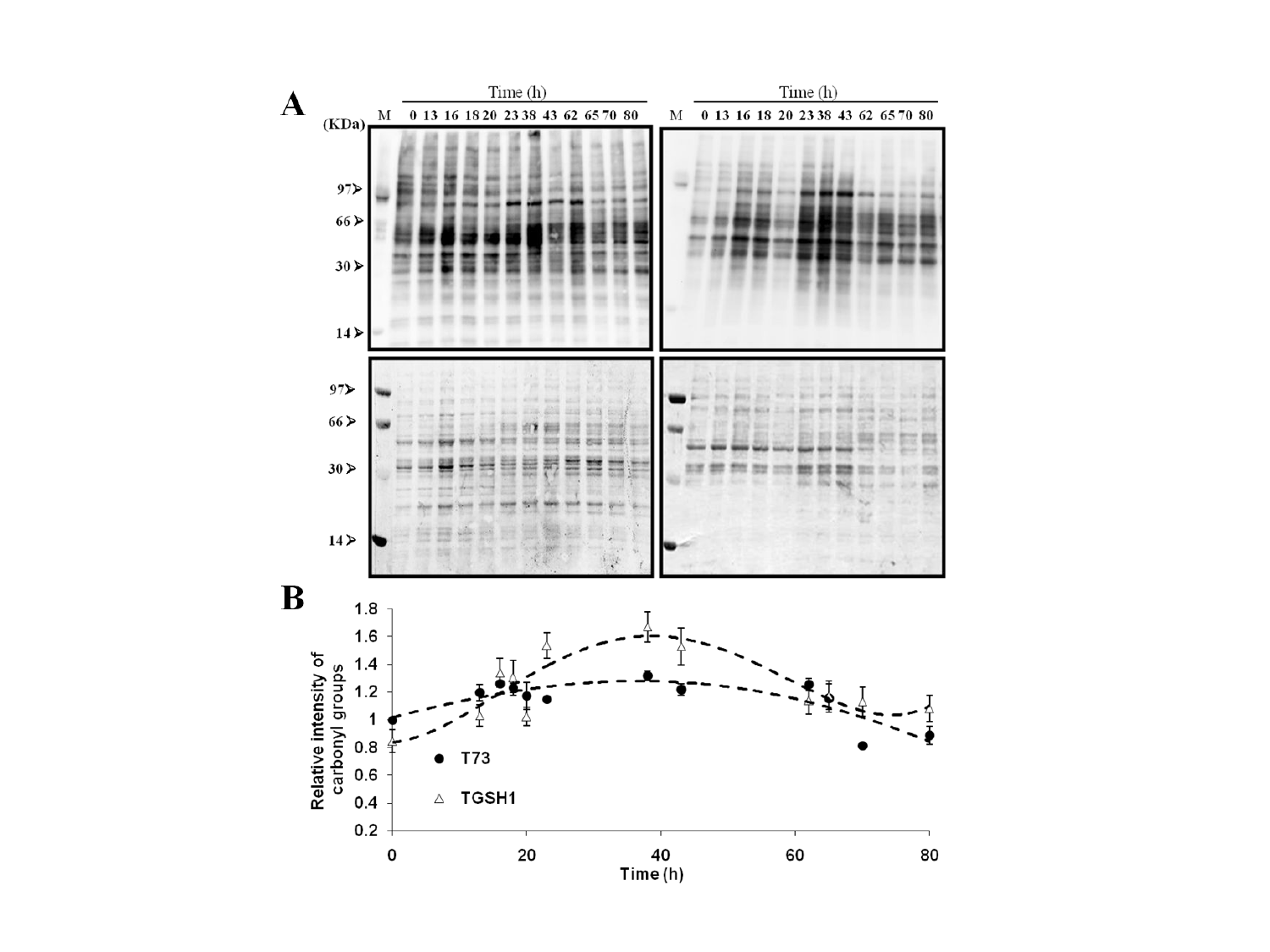

Supplement: Additional file 2 — Protein carbonylation along bench-top trials of biomass propagation for T73 and TGSH1 strain. Western analysis of oxidatively damaged proteins (top panels) and total protein stain (bottom panels) for T73 (left panels) and TGSH1 (right panels) strains (A). Panel B shows quantification of protein carbonyl content of T73 (black circles) and TGSH1 (open squares) data shown in panel A. Data was normalized to total protein in Coomassie stained gels. In order to avoid technical errors due to different exposure times, comparison between both strains was carried out in the same experiment. The mean of three independent experiments and standard deviations are shown. Protein carbonylation in samples from both strains between 0 and 65 h were significantly different with p < 0.01, and samples at 70 and 80 h were significantly different with p < 0.05. [file 1475-2859-9-9-S2.PPT]
